# Safety, feasibility and effects of an individualised walking intervention for women undergoing chemotherapy for ovarian cancer: a pilot study

**DOI:** 10.1186/1471-2407-11-389

**Published:** 2011-09-08

**Authors:** Melissa J Newton, Sandi C Hayes, Monika Janda, Penelope M Webb, Andreas Obermair, Elizabeth G Eakin, David Wyld, Louisa G Gordon, Vanessa L Beesley

**Affiliations:** 1 School of Public Health, Queensland University of Technology, Brisbane, Australia; 2 Gynaecological Cancers Group, Queensland Institute of Medical Research, Brisbane, Australia; 3 Queensland Centre for Gynaecologic Oncology, Royal Brisbane and Women's Hospital, Brisbane, Australia; 4 School of Population Health, The University of Queensland, Brisbane, Australia; 5 Department of Medical Oncology, Royal Brisbane and Women's Hospital, Brisbane, Australia; 6 Centre for Applied Health Economics, Griffith University, Brisbane, Australia

**Keywords:** Ovarian neoplasm, exercise, chemotherapy, feasibility

## Abstract

**Background:**

Exercise interventions during adjuvant cancer therapy have been shown to increase functional capacity, relieve fatigue and distress and may assist rates of chemotherapy completion. These studies have been limited to breast, gastric and mixed cancer groups and it is not yet known if a similar intervention is even feasible among women with ovarian cancer. We aimed to assess safety, feasibility and potential effect of a walking intervention in women undergoing chemotherapy for ovarian cancer.

**Methods:**

Women newly diagnosed with ovarian cancer were recruited to participate in an individualised walking intervention throughout chemotherapy and were assessed pre- and post-intervention. Feasibility measures included session adherence, compliance with exercise physiologist prescribed walking targets and self-reported program acceptability. Changes in objective physical functioning (6-minute walk test), self-reported distress (Hospital Anxiety and Depression Scale), symptoms (Memorial Symptom Assessment Scale - Physical) and quality of life (Functional Assessment of Cancer Therapy - Ovarian) were calculated, and chemotherapy completion and adverse intervention effects recorded.

**Results:**

Seventeen women were enrolled (63% recruitment rate). Mean age was 60 years (SD = 8 years), 88% were diagnosed with FIGO stage III or IV disease, 14 women underwent adjuvant and three neo-adjuvant chemotherapy. On average, women adhered to > 80% of their intervention sessions and complied with 76% of their walking targets, with the majority walking four days a week at moderate intensity for 30 minutes per session. Meaningful improvements were found in physical functioning, physical symptoms, physical well-being and ovarian cancer-specific quality of life. Most women (76%) completed ≥85% of their planned chemotherapy dose. There were no withdrawals or serious adverse events and all women reported the program as being helpful.

**Conclusions:**

These positive preliminary results suggest that this walking intervention for women receiving chemotherapy for ovarian cancer is safe, feasible and acceptable and could be used in development of future work.

**Trial registration:**

ACTRN12609000252213

## Background

Ovarian cancer is the second most common gynaecologic malignancy [1] with treatment typically involving radical cytoreductive surgery and chemotherapy. Women with ovarian cancer differ from other cancer groups due to their older age, predominantly late stage diagnosis, poorer prognosis, treatment involving major abdominal surgery and different chemotherapy agents/doses/regimens with significant side-effects [2]. Overall, up to 90% of women receiving chemotherapy for ovarian cancer experience nausea, difficulty sleeping and pain [3], and the presence of these side effects has been associated with reduced physical function [4] and/or premature chemotherapy cessation [5]. Anxiety and depression are also common during treatment for ovarian cancer, with higher rates than those reported in other cancer cohorts [6]. The presence of these physical and psychological concerns likely contribute to low levels of quality of life (QoL) observed during chemotherapy for ovarian cancer [7] and may be associated with reduction of planned chemotherapy dose [8] and overall survival [7].

In a previous study, up to one-third of women receiving treatment for ovarian cancer were not able to complete their prescribed first-line chemotherapy course; specifically, approximately 10% required a dose reduction, replacement or removal of an agent and 6% ceased treatment altogether [3]. Intravenous chemotherapy completion rates using cisplatin and paclitaxel in advanced stage ovarian cancer have been reported to range between 83% [9] and 86% [10]. Others observed changes to chemotherapy schedules in 56% to 91% of colon or breast cancer patients [11,12]. This is important because, among patients with breast cancer changes to their intended chemotherapy courses or doses reduced response rates to treatment from 68% to 30% [13].

Exercise has been suggested as a means of attenuating physical and psychological side-effects of cancer treatment. A recent meta-analysis involving 33 studies of exercise intervention studies conducted during cancer treatment found that the majority of interventions resulted in improved physical activity, aerobic fitness, and QoL, as well as reductions in anxiety, body fat percentage, and body weight [14]. The interventions evaluated commonly involved aerobic or combined aerobic/resistance training (n = 29), at a moderate to vigorous intensity (n = 20), three to five times per week (n = 21), for a duration of 20-45 minutes per session (n = 18), and for at least five weeks. The majority of the studies (n = 26) enrolled women with breast cancer, with only one study including women with ovarian cancer. The potential for exercise to influence chemotherapy completion rates has been considered in only one study to date [15]. Women were assigned to a supervised aerobic exercise intervention (three times per week, 15-45 minutes duration, 60-80% VO_2_max) or supervised resistance training intervention (three times per week performing 8-12 repetitions for two sets of nine different exercises) conducted throughout the duration of chemotherapy, respectively. Compared to standard care (66%), 74% and 78% of women in the intervention groups completed their prescribed chemotherapy. These results suggest that exercise during chemotherapy may improve treatment adherence, and are worthy of further investigation.

The benefits of individualised exercise programs for all cancer patients in minimising risk of injury and optimising individual gain has been highlighted in the Australian Exercise and Sport Science Association position stand on optimising cancer outcomes through exercise [16]. To date, women with ovarian cancer have not been the focus of randomised controlled trials involving exercise intervention. A recent publication reported on the feasibility of a lifestyle counseling intervention, where women with ovarian cancer were given generic advice to stay as active as their energy level allowed during chemotherapy (n = 27)[17]. However, there have been no published individualised exercise intervention studies involving a heterogeneous group of women with ovarian cancer. The main purpose of this study was therefore to assess the safety and feasibility of an individualised walking intervention in women undergoing chemotherapy treatment for ovarian cancer. We also describe pre-post intervention changes in physical functioning, anxiety, depression, physical symptoms and QoL, and record chemotherapy completion rates.

## Methods

This non-randomised phase 2 trial assessed safety and feasibility of a walking intervention prior and after the completion of chemotherapy. Pre-intervention assessment was conducted prior to the first or second cycle of neo-adjuvant or adjuvant chemotherapy, while the post-intervention assessment was conducted three weeks after the last dose of chemotherapy. Ethics approval was obtained from Queensland Institute of Medical Research (P1222), Queensland University of Technology (0900000333) and Royal Brisbane and Women's Hospital (HREC/08/QRBW/19).

Eligible patients were those living in Queensland, Australia and treated at Royal Brisbane and Women's Hospital for invasive ovarian, peritoneal or fallopian tube cancer between June 2009 and September 2010. They were about to start their first or second cycle of adjuvant or neo-adjuvant chemotherapy; aged 18 years or older; able to complete questionnaires in English and provide informed consent. Women who were identified by their gynaecological oncologist as too sick to participate or who had a prior malignancy (excluding non-melanoma skin cancers) within the last five years were excluded. Under the original study protocol, where intervention delivery was restricted to face-to-face contact, 16 out of 28 women (57%) were ineligible because they lived > 60 kms from the hospital, significantly influencing our ability to recruit women into the study. Consequently, the mode by which the intervention could be delivered was broadened to allow for telephone-delivery and as such the residence criterion was removed.

Prior to the commencement of the intervention, demographic data including age, marital status, education and health insurance status (public or private), were obtained from consenting women via self-report. Clinical information on stage, histology and chemotherapy regimen (according to the classifications used by the International Federation of Gynecology and Obstetrics FIGO) were abstracted from the medical records.

Participants were given an educational booklet related to the walking intervention, which included topics such as how to monitor walking intensity, when not to walk, and monitoring changes in treatment-related side-effects. Due to the nature of the study population, the weekly walking prescription (frequency, intensity and duration) was individualised by an exercise physiologist (allied health professional with tertiary training in exercise science and with additional specialist training in exercise prescription for cancer patients). Initial targets were based on pre-intervention assessment of physical functioning and level of physical activity. Sedentary women were instructed to begin by taking frequent (most days), but lower-intensity, shorter duration (10 minute) walks. Active women were initially instructed to maintain their current number of sessions and increase firstly the duration and later the intensity. All participants were instructed to record details of their walking activity using a logbook. Participants met with the exercise physiologist once per week, either face-to-face (for women living within 60 km of the treating hospital) or over the telephone (for regional/rural women) for the duration of their chemotherapy treatment. For the face-to-face group this session included a supervised walk with the exercise physiologist. During these weekly sessions, presence and change to treatment-related side-effects were identified, barriers to walking were discussed and resolved when possible, details of previous week's walking sessions were discussed, and based on this the subsequent week's walking targets were prescribed per individual. The weekly session duration with the exercise physiologist was between 20-60 minutes, with longer sessions typically required in the earlier weeks of the intervention to ensure the topics within the education booklet were discussed. Given the pilot nature of this work, the mode of contact with the exercise physiologist was flexible. For example, telephone contact was allowed for local women when face-to-face sessions were difficult to arrange and face-to-face contact was provided to rural women when they returned to hospital for follow-up care or treatment.

Recruitment rate was defined by the number of consenting, divided by the number of eligible women approached to participate. Retention was determined by the number of participants who completed the pre- and post-intervention testing. Adherence was ascertained by the number of completed sessions relative to the number of scheduled sessions with the exercise physiologist, while compliance was determined by comparing exercise physiologist prescribed walking duration, frequency and intensity with details recorded in participants' completed walking logs. Acceptability of the walking program was assessed post-intervention using a written evaluation form, which asked participants to rate on a seven-point Likert-scale how helpful the program, the education booklet, and the exercise physiologist sessions were. Participants were also asked to indicate how they felt about participating in the walking program during their chemotherapy treatment. Participant-reported adverse effects (safety) were pre-defined as any unfavorable change from 'normal' condition that limited everyday living.

*Physical functioning *was measured using the six-minute walk test (6MWT), which has strong test-retest reliability (intraclass correlation = 0.97) [18]. The test requires participants to walk along a flat, 10-metre length, straight pathway for six continuous minutes at a comfortable pace. Distance travelled by the test cessation was recorded. *Anxiety and depression *were measured by the validated Hospital Anxiety and Depression Scale (HADS) [19]. The HADS is a self-rated instrument containing two subscales, anxiety and depression (coefficient alphas of 0.93 and 0.90, respectively), with seven items per subscale, using a 4-point Likert scale. *Symptoms *were measured using the physical symptom subscale of the validated Memorial Symptom Assessment Scale (MSAS-PHYS)(Cronbach's alpha = 0.88) [20]. This scale evaluates frequency, severity and distress of 12 treatment-related symptoms over the previous seven days. Frequency and distress are rated on a 5-point Likert scale, while the severity dimension is rated on a 4-point Likert scale, ranging from one to four. *Quality of life *was measured using the Functional Assessment of Cancer Therapy - Ovary (FACT-O) [21]. The FACT-O includes 39 items to measure five wellbeing domains (physical, social/family, emotional, functional and ovarian cancer-specific concerns) over the past seven days, on a 5-point Likert scale. This instrument has undergone extensive reliability and validity testing showing that internal consistency and test-retest reliability is adequate [21]. The HADS, MSAS-PHYS and FACT-O were all participant-administered. *Chemotherapy completion *information was abstracted from medical records by the research nurse. As is convention, the relative dose intensity (RDI) of each chemotherapy agent was calculated by expressing the total delivered dose per unit time as a percentage of the initial target dose, then each agent (commonly two agents prescribed) was added together and divided by the number of agents to get a total RDI [22].

We pre-defined acceptable walking intervention adherence as women participating in 75% or more of the scheduled weekly sessions with the exercise physiologist. Participants were considered compliant to the prescribed walking intervention when they achieved two out of three exercise prescription targets (frequency, intensity and duration). Pre- and post-intervention continuous outcomes (physical functioning, anxiety, depression, symptoms and QoL) were summarised using median and ranges scores. Pre- and post-intervention data were compared statistically using Wilcoxon Signed Ranked tests, with statistical significance set at p < 0.05. Results were analysed using SPSS 16.0. *A priori *defined clinically important changes (±) in outcomes were as follows: ≥ 54 meters for the 6MWT [23], 1.5 points for HADS scores [24], 0.2 for MSAS-PHYS score [25], 2 for the FACT-O subscales, 3 for ovarian cancer-specific concerns and 5 for the overall FACT-O scale [26]. Predefined acceptable chemotherapy completion was based on a previously applied criterion of 85% or more of initially planned chemotherapy dose [15].

## Results

### Recruitment

**Figure 1:**
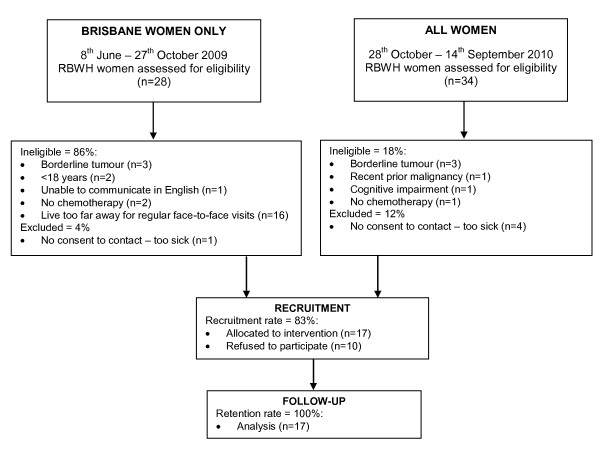
**Participant recruitment, allocation and retention**.

Sixty-two women presenting with possible ovarian cancer at the participating hospital were screened over approximately 15 months (Figure 1). Nineteen women were ineligible as a consequence of having borderline tumors (n = 6), not receiving chemotherapy (n = 3), being considered too sick to participate by their gynaecologist (n = 5) or other reasons (n = 5). Of the 27 eligible women, 17 (63%) consented to participate, eight of whom lived locally and therefore received weekly contacts with the exercise physiologist on a face-to-face basis, while the remaining nine women, received their weekly contacts over the telephone.

### Sample characteristics

Participant's pre-intervention demographic and clinical characteristics are summarised in Table 1. Women were aged between 44-71 years (mean 60.4 years). Approximately half (58%) were married or in a de facto relationship, all had at least a high school education, and most (76%) did not have private health insurance. The majority of women were diagnosed with a serous cell type on histological diagnosis (82%) and FIGO stage III or IV disease (88%). Prescribed chemotherapy varied; the most common regimen being six times three-weekly cycles of carboplatin and paclitaxel (59%). Chemotherapy was administered intravenously (with or without the addition of intraperitoneal delivery) with three women receiving neo-adjuvant therapy and 14 adjuvant chemotherapy. Compared to women with ovarian cancer listed in the population-based gynaecological cancer registry (n = 1,286) [27], in this pilot study, there was a higher proportion of women in the 60-69 year age bracket (47% compared with 23%), a higher proportion of women with FIGO stage III or IV disease (88% compared with 50%), and all women received surgery and chemotherapy compared to only 75% of women from the registry.

**Table 1:** Demographic and clinical characteristics of participants at baseline assessment (n = 17)

Characteristics		
*Demographic*		
Age at baseline, mean (SD)	60 (8)	
	
	**N**	**%**
	
Marital status		
Never married	3	18
De facto/married	10	58
Separated/divorced	3	18
Widowed	1	6
Education level		
University	2	12
Technical/trade	2	12
Secondary (grade 10-12)	13	76
Private health insurance		
No	13	76
Yes	4	24
Body mass index categories*		
Underweight (< 18.5 kg/m^2^)	2	20
Healthy weight (18.5-24.9 kg/m^2^)	4	40
Overweight (25-29.9 kg/m^2^)	2	20
Obese (30+ kg/m^2^)	2	20
*Clinical*		
Primary cancer site		
Ovary	13	76
Peritoneum	4	24
Histology		
Endometrioid	1	6
Serous and other	14	82
Mucinous	1	6
Unknown	1	6
Disease stage (FIGO)		
I	1	6
II	1	6
III	11	64
IV	4	24
Chemotherapy regimen		
6 × 3-weekly Carboplatin + Paclitaxel	10	59
Other	7	41
Chemotherapy route		
Intravenous	9	53
Intravenous + Intraperitoneal	8	47
Chemotherapy course		
Adjuvant	14	82
Neo-adjuvant	3	18

Body mass index not collected for seven regional women due to inaccessibility;*

Not known; NK

International Federation of Gynecology and Obstetrics; FIGO

### Feasibility and safety

No withdrawals were recorded during the walking intervention (100% retention). The number of weeks under active chemotherapy (and therefore possible exercise physiologist sessions) ranged from 11 to 21 weeks. Overall group adherence was 90% (range 55% to 100%), with 14 of 17 women (82%) participating in at least 75% of scheduled face-to-face or telephone intervention sessions. Participants missed a median of two sessions (range 0-8), most commonly due to cancer treatment-related hospitalization, holidays, or being too ill. In addition, for Brisbane women (n = 8), 17 out of 136 face-to-face sessions were changed to telephone sessions (each local woman had one to five sessions over the telephone), while one of the nine women living more than 60 km from hospital had 11 face-to-face sessions as they coincided with treatment.

**Figure 2:**
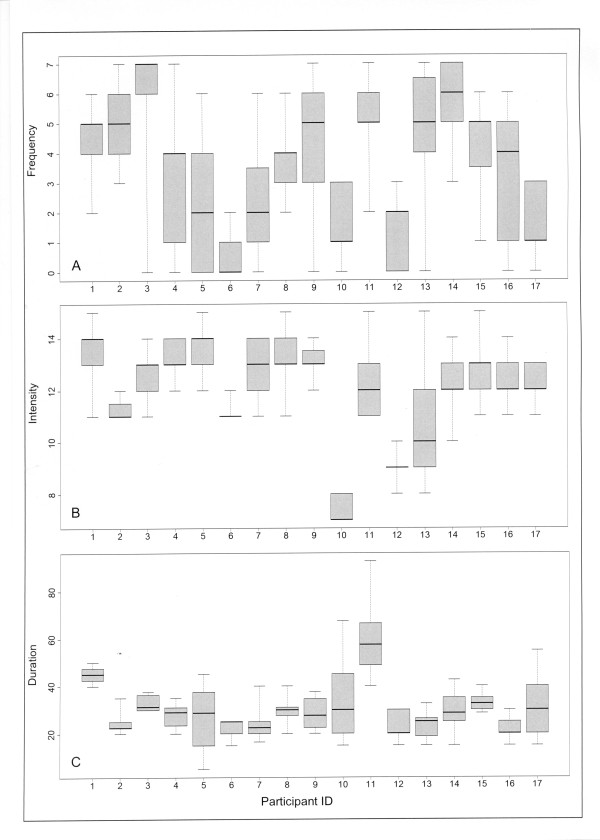
**Box plots of frequency, intensity and duration of walking achieved throughout the intervention for each participant (assessed by self-report exercise log; n = 17)**. Figure 2 (A): Frequency (number of days) per week. Figure 2 (B): Intensity level (rating of perceived exertion scale) per session. Figure 2 (C): Duration (minutes) of walking per session. Footnote: Symbols: box = 1^st ^& 3^rd ^quartiles, line inside box = median, whisker length = minimum & maximum (range).

The median and range of frequency (number of days), intensity (rating of perceived exertion scale) and duration (minutes) of walking sessions undertaken each week for each woman are illustrated in Figure 2. On average women walked four days a week (range 0-7) at moderate intensity for 30 minutes per session (range 22-57 minutes). Overall, compliance with at least two out of three individual weekly prescription targets (frequency, intensity and duration) ranged from 42% to 94%, with a median of 88%.

Sixteen women (94%) completed and returned the intervention evaluation, all of whom found the program to be either 'helpful' or 'very helpful'. The vast majority (81%) rated the sessions with the exercise physiologist as 'very helpful', while 75% considered the program to be 'excellent'. One minor adverse event, a fall causing minor cuts and bruises, occurred (no major adverse event).

### Clinical effects

Clinically important improvements were observed in physical functioning, physical symptoms, physical and ovarian-specific QoL between pre- and post-intervention (Table 2). Improvements were also observed in seven out of twelve physical symptoms including lack of appetite, pain, feeling drowsy, constipation, dry mouth, nausea and weight loss. No change was found in anxiety or depression or in the social, emotional and functional wellbeing QoL subscales.

**Table 2:** Outcome measures assessed pre- and post-participation in the walking intervention (n = 17)

Outcome	Baseline	Follow-up	
	**Median** **(min, max)**	**Median** **(min, max)**	**P-value^a^**

Physical functioning^b^	332 (266, 356)	395 (356, 460)*	0.01
Anxiety	4 (1, 15)	4 (0, 16)	0.63
Depression	3 (0, 16)	4 (0, 13)	0.16
Physical symptoms	1.06 (0.0, 2.33)	0.60 (0.06, 2.06)*	0.14
Quality of life^c ^(FACT-O)	109 (72, 146)	113 (67, 148)	0.10
Physical wellbeing	18 (12, 27)	23 (12.0, 28)*	0.08
Social wellbeing	23 (12, 28)	22 (8, 28)	0.11
Emotional wellbeing	20 (7, 24)	21 (10, 24)	0.29
Functional wellbeing	19 (7, 28)	19 (7, 28)	0.02
Ovarian-specific concerns	31 (20, 41)	36 (21, 44)*	0.04

Clinically meaningful change;*

Wilcoxon signed ranked test used for analysis;^a^

Physical functioning is measured in metres (m)

Physical functioning data not collected for seven regional women due to inaccessibility;^b^

A higher score indicates a better quality of life;^c^

Functional Assessment of Cancer Therapy-Ovary; FACT-O

All women received all of their planned chemotherapy cycles. Relative dose intensity received of planned chemotherapy ranged from 67% to 100% (median 92%). Thirteen out of 17 (76%) women had an RDI equal to or above 85% of their scheduled dose.

## Discussion

Importantly, results suggest participating in an individually tailored face-to-face or telephone delivered walking program during chemotherapy for ovarian cancer is safe and feasible. Retention (100%), adherence (82%) and compliance rates (88%) in this pilot were high and compare favorably with feasibility data from previous studies of home-based exercise conducted with patients undergoing chemotherapy for other cancers (adherence 60-90%) [4,28]. Our high rates may have been related to the close monitoring (weekly sessions with the exercise physiologist), personalised feedback, or on-going support provided by the exercise physiologist. The home-based nature of the intervention, allowing the participants to undertake sessions at times most convenient to them may also have contributed. In addition, and in contrast to most other interventions, our moderate exercise was achieved through walking, reported to be the preferred type of aerobic-exercise for women with ovarian cancer [29].

While national and international evidence-based guidelines on the participation in exercise during and following treatment for cancer exist, few previous exercise interventions have involved women with ovarian cancer. As a consequence, it was a considerable challenge to mount this pilot study, given the prevailing assumption that exercise intervention is not appropriate for women undergoing chemotherapy for ovarian cancer. The majority of women in this study walked on average four days a week, at moderate intensity, for approximately 30 minutes per session, throughout the duration of their chemotherapy. Treatment-related side-effects presented barriers to participating in the walking intervention, however, the participant and exercise physiologist together identified ways in which these could be overcome. For example, the walking route was modified to ensure symptoms such as diarrhea or nausea could be accommodated. Importantly, not only were woman able to participate fully in the walking intervention, but they also found the program highly acceptable.

Participating in the walking intervention was associated with improvements in physical functioning, physical symptoms and ovarian cancer-specific QoL. While the absence of a randomised control group prevents the establishment of a causal relationship and the small sample size means that some changes did not reach statistical significance, the changes observed are similar to those reported from aerobic exercise interventions in women with breast cancer [28]. Further, results from cancer studies suggest that patients typically decline in physical function and QoL during chemotherapy, and report symptoms of high frequency and severity [30]. Therefore, it seems plausible that a walking intervention during chemotherapy may prevent declines and reduce the impact of symptoms.

Three quarters (n = 13) of the women in this study received greater than or equal to 85% of their planned chemotherapy dose (median 92%) and all women completed all of their planned cycles. It is difficult to compare these data with other reports of chemotherapy completion in ovarian cancer as previous studies have been drug trials that deliver non-standard chemotherapy regimens and limit their samples to women with advance disease [9,10]. As such, a larger sample with randomised controlled data is needed to confirm whether exercise can assist in improving chemotherapy completion rates.

## Conclusion

This is the first individualised exercise intervention study that focuses entirely on women with ovarian cancer whilst undergoing chemotherapy. Although this is a non-randomised study with a relatively small sample size, important preliminary findings have been derived from this work. Despite the intervention being conducted during a time typically associated with elevated distress and treatment side-effects that are often severe enough to alter or cease chemotherapy prescription, women perceived the program as helpful. Most importantly, the program was safe and feasible and showed promise for reducing some symptoms, and optimising physical function and QoL during chemotherapy. These results provide the necessary platform from which a larger, randomised controlled trial can be developed.

## Competing interests

The authors declare that they have no competing interests.

## Authors' contributions

MN, VB and SH had full access to all the data in the study and take responsibility for the integrity of the data and accuracy of data analysis. VB, SH, MJ, PW, EE, LG and DW initiated study concept and design. In addition clinician, AO, specialises in gynaecological oncology and enabled participant recruitment. Data acquisition was conducted by MN. Analysis and interpretation of data was carried out by MN, SH, VB and MJ. MN, SH, VB and MJ drafted the manuscript and AO, PW, EE, LG and DW provided critical revisions and important intellectual content. Study supervision and funding was supplied by SC, MJ, VB and PW. All authors read and approved the final manuscript.

## Pre-publication history

The pre-publication history for this paper can be accessed here:

http://www.biomedcentral.com/1471-2407/11/389/prepub

## References

[B1] Australian Institute of Health and Welfare (AIHW) & Australasian Association of Cancer Registries (AACR) Cancer in Australia 2000 2003 Canberra: AIHW (Cancer Series no 23) AIHW Cat. No. CAN 18

[B2] Quasthoff S Hartung H Chemotherapy-induced peripheral neuropathy J Neurol 2002 249 9 17 10.1007/PL00007853 11954874

[B3] Gordon LG Scuffham PA Beesley VL Green AC DeFazio A Wyld DK Clavarino AM Australian Ovarian Cancer Study Group Webb PM Outcomes and related medical costs for women with ovarian cancer in Australia: a patient-level analysis over 21/2 years Int J Gynecol Cancer 2010 20 757 765 10.1111/IGC.0b013e3181dbd13f 20973265

[B4] Schwartz A Physical activity after a cancer diagnosis: psychosocial outcomes Cancer Invest 2004 22 1 82 92 10.1081/CNV-120027582 15069765

[B5] Olsen CM Bain CJ Jordan S Nagle C Green A Whiteman D Webb P Australian Cancer Study (Ovarian Cancer) and Australian Ovarian Cancer Study Group Recreational Physical Activity and Epithelial Ovarian Cancer: A Case-Control Study, Systematic Review, and Meta-analysis Cancer Epidemiol Biomarkers Prev 2007 16 11 2321 2330 10.1158/1055-9965.EPI-07-0566 18006921

[B6] Bodurka-Bevers D Basen-Engquist K Carmack C Fitzgerald M Wolf J de Moor C Gershenson D Depression, Anxiety, and Quality of Life in Patients with Epithelial Ovarian Cancer Gynecol Oncol 2000 78 302 308 10.1006/gyno.2000.5908 10985884

[B7] Wenzel L Huang H Armstrong D Walker J Cella D Health-related quality of life during and after intraperitoneal versus intravenous chemotherapy for optimally debulked ovarian cancer: a Gynecologic Oncology Group Study J Clin Oncol 2007 25 437 443 10.1200/JCO.2006.07.3494 17264340

[B8] Oken MM Creech RH Tormey DC Horton J Davis T McFadden ET Carbone P Toxicity and response criteria of the Eastern Cooperative Oncology Group Am J Clin Oncol 1982 5 649 655 10.1097/00000421-198212000-00014 7165009

[B9] Markman M Bundy B Alberts D Clark-Pearson DL Carson LF Wadler S Sickel J Phase III Trial of Standard-Dose Intravenous Cisplatin Plus Paclitaxel Versus Moderately High-Dose Carboplatin Followed by Intravenous Paclitaxel and Intraperitoneal Cisplatin in Small-Volume Stage III Ovarian Carcinoma: An Intergroup Study of the Gynecologic Oncology Group, Southwestern Oncology Group, and Eastern Cooperative Oncology Group J Clin Oncol 2001 19 4 1001 1007 11181662 10.1200/JCO.2001.19.4.1001

[B10] Armstrong DK Bundy B Wenzel L Huang H Baergen R Lele S Copeland L Walker J Burger R for the Gynecologic Oncology Group Intraperitoneal cisplatin and paclitaxel in ovarian cancer New Eng J Med 2006 354 1 34 43 10.1056/NEJMoa052985 16394300

[B11] Hirria A Leung D Trainor K Borgen P Norton L Hudis C Factor influencing treatment patterns of breast cancer patients age 75 and older Crit Rev Oncol Hematol 2003 46 121 126 10.1016/S1040-8428(02)00133-6 12711357

[B12] Law CC Fu YT Postoperative adjuvant 5-Fluororuacil plus levamisole chemotherapy for stage III colon carcinoma: 7-year experience in a single institution J Hong Kong College Rad 2002 5 97 104

[B13] D'hondt R Paridaens R Wildiers H Pauwelyn K Thomas J Dumez H Van Oosterom A Safety and efficacy of weekly docetaxel in frail and/or elderly patients with metastatic breast cancer: a phase II study Anticancer Drug 2004 15 341 346 10.1097/00001813-200404000-00005 15057137

[B14] Speck RM Courneya KS Mâsse LC Duval S Schmitz KH An update of controlled physical activity trials in cancer survivors: a systematic review and meta-analysis J Cancer Surviv 2010 4 87 100 10.1007/s11764-009-0110-5 20052559

[B15] Courneya KS Segal RJ Mackey JR Gelmon K Reid R Friedenreich C Ladha A Proulx C Vallance J Lane K Yasui Y McKenzie D Effects of aerobic and resistance exercise in breast cancer patients receiving adjuvant chemotherapy: a multicenter randomized controlled trial J Clin Oncol 2007 25 4396 4404 10.1200/JCO.2006.08.2024 17785708

[B16] Hayes SC Spence RR Galvao DA Newton R Australian Association for Exercise and Sport Science position stand: optimising cancer outcomes through exercise J Sci Med Sport 2009 12 428 434 10.1016/j.jsams.2009.03.002 19428291

[B17] Von Gruenigen V Frasure H Kavanagh M Lerner E Waggoner SE Courneya KS Feasibility of a lifestyle intervention for ovarian cancer patients receiving adjuvant chemotherapy Gynecol Oncol 2011 in press 10.1016/j.ygyno.2011.04.043 21600635

[B18] ATS statement: guidelines for the six-minute walk test Am J Respir Crit Care Med 2002 166 111 117 12091180 10.1164/ajrccm.166.1.at1102

[B19] Zigmond AS Snaith RP The hospital anxiety and depression scale Acta Psychiatr Scand 1983 67 6 361 370 10.1111/j.1600-0447.1983.tb09716.x 6880820

[B20] Chang V Hwang S Feuerman M Kasimis B Thaler H The Memorial Symptom Assessment Scale Short Form (MSAS-SF) Validity and Reliability Cancer 2000 89 1162 1171 10.1002/1097-0142(20000901)89:5<1162::AID-CNCR26>3.0.CO;2-Y 10964347

[B21] Basen-Engquist K Bodurka-Bevers D Fitzgerald M Webster K Cella D Hu S Gershenson D Reliability and validity of the functional assessment of cancer therapy - Ovarian J Clin Oncol 2001 19 1809 1817 11251013 10.1200/JCO.2001.19.6.1809

[B22] Terada Y Nakamae H Aimoto R Kanashima H Sakamoto E Aimoto M Inoue E Koh H Nakane T Takeoka Y Ohsawa M Koh KR Yamane T Nakao Y Ohta K Mugitani A Teshima H Hino M Impact of relative dose intensity (RDI) in CHOP combined with rituximab (R-CHOP) on survival in diffuse large B-cell lymphoma J Exper Clin Can Res 2009 28 116 10.1186/1756-9966-28-116 PMC2743657 19689822

[B23] Rasekaba T Lee AL Naughton MT Williams TJ Holland AE The six-minute walk test: a useful metric for the cardiopulmonary patient Intern Med J 2009 39 8 495 501 10.1111/j.1445-5994.2008.01880.x 19732197

[B24] Puhan M Frey M Büchi S Schünemann H The minimal important difference of the hospital anxiety and depression scale in patients with chronic obstructive pulmonary disease Health Qual Life Outcome 2008 6 46 10.1186/1477-7525-6-46 PMC2459149 18597689

[B25] Walker P Which symptoms are prevalent in patients with recurrent ovarian cancer? J Clin Oncol 2008 26 No 15S 20689

[B26] Sloan JA Cella D Hays RD Clinical significance of patient-reported questionnaire data: another step toward consensus J Clin Epidemiol 2005 58 12 1217 1219 10.1016/j.jclinepi.2005.07.009 16291464

[B27] Queensland Centre for Gynecological Cancer Outcome data statistical report 2008

[B28] Courneya KS Friedenreich CM Sela RA Quinney HA Rhodes RE Handman M The group psychotherapy and home-based physical exercise (Group-Hope) trail in cancer survivors: Physical fitness and quality of life outcomes Psycho-oncology 2003 12 357 374 10.1002/pon.658 12748973

[B29] Stevinson C Capstick V Schepansky A Tonkin K Vallance JK Ladha AB Steed H Faught W Courneya KS Physical activity preferences of ovarian cancer survivors Psycho-oncology 2009 18 4 422 428 10.1002/pon.1396 19243089

[B30] Joly F Vardy J Pintilie M Tannock I Quality of life and/or symptom control in randomized clinical trials for patients with advanced cancer Ann Oncol 2007 18 1935 1942 10.1093/annonc/mdm121 17698837

